# Quantitative proteomic analysis of sperm in unexplained recurrent pregnancy loss

**DOI:** 10.1186/s12958-019-0496-5

**Published:** 2019-07-09

**Authors:** Dena Xue, Yi Zhang, Yixin Wang, Jun Wang, Fengxiao An, Xiaowei Sun, Zhenhai Yu

**Affiliations:** 1 Center for Reproductive Medicine, Shandong Provincial Hospital Affiliated to Shandong University, Jinan, 250001 Shandong China; 2 Department of Reproductive Medicine, Affiliated Hospital of Weifang Medical University, Weifang, Shandong Province, People’s Republic of China

**Keywords:** Recurrent pregnancy loss, iTRAQ, Sperm, Biomakers, Gene functions and pathways

## Abstract

**Background:**

Recurrent pregnancy loss (RPL) refers to two or more spontaneous abortions that occur consecutively with the same spouse. RPL severely affects human reproduction health, and causes extreme physical and mental suffering to patients and their families.

**Methods:**

We used isobaric tags for relative and absolute quantitation (iTRAQ), which was coupled with liquid chromatography mass spectrometry (LC-MS) proteomic analysis, in order to identify differentially expressed proteins. Moreover, we used western blot to analyze differentially expressed proteins.

**Results:**

Of the 2350 non-redundant proteins identified, 38 proteins were significantly altered and were identified as potential biomarkers for RPL. The protein-protein interaction network constructed using GeneMANIA revealed that 35.55% displayed similar co-expression, 30.87% were predicted, and 20.95% had physical interaction characteristics. Based on Gene ontology classification and KEGG pathway enrichment analyses, the majority of these differentially expressed proteins were found to be related to biological regulation, metabolic and cellular processes, protein binding and different enzymes activities, as well as disorder of fat and glucose metabolic pathways. It is noteworthy that three metabolism related biomarkers (HK1, ACLY, and FASN) were further confirmed through western blot analysis.

**Conclusions:**

These results suggest that these differentially expressed proteins may be used as biomarkers for RPL, and related signaling pathways may play crucial roles in male induced RPL.

**Electronic supplementary material:**

The online version of this article (10.1186/s12958-019-0496-5) contains supplementary material, which is available to authorized users.

## Background

Recurrent pregnancy loss (RPL) refers to the consecutive occurrence of two or more spontaneous abortions with the same spouse in early pregnancy [1]. The incidence of RPL accounts for about 1% of all pregnancies and causes extreme physical and mental suffering to patients and their families [1]. Although many factors have been investigated, including chromosomal, endocrine and anatomical aberrations, prethrombotic state, immune disorders and infections, the causes in the half of RPL cases are still unknown which are labeled as unexplained [2]. Because the male contributes 50% of the DNA to the embryo, it is not surprising that paternal factors could also contribute to pregnancy loss [3]. The association is first studied between diminished DNA content in sperm and spontaneous abortion with special reference to male factor, which was published in 1966 [4]. Furthermore, another study also examines the relationship between recurrent pregnancy loss and sperm characteristics [5]. However, researches on RPL mainly focus on maternal factors, the male contributions have been largely unexamined and remain poorly understood [6, 7].

Advances in technological developments are expected to find new biomarkers for RPL. As an emerging research tool in the post-genome era, proteomics can be utilized for studying the protein expression, function and protein-protein interactions [8]. It has become one of the most important tools for studying sperm protein profiles. An in-depth understanding of the sperm proteomics could be conductive to explain the roles of sperm proteins in how to cause RPL. Significant advances in methods and strategies related to proteomics, including mass spectrometry and liquid chromatography, have enabled the analysis of thousands of complex cellular proteins present in sperm [9, 10]. A recent study compared the differences in the sperm proteome by liquid chromatography-tandem mass spectrometry and found that the proteins associated with sperm function and fertilization process were compromised in testicular cancer patients [11]. Up to now, there is no report of investigation the difference in the sperm proteome of males for RPL at a proteomic level.

In this study, the sperm proteome is analyzed to elucidate the proteomic expression profiles of sperm, and to further ascertain the abnormal protein biomarkers of males for RPL. We perform isobaric tags for relative and absolute quantitation (iTRAQ) to analyze sperm proteomic changes in RPL patients. We aim to uncover significantly altered sperm proteins and pathways in RPL.

## Materials and methods

### Ethics statement

All procedures were carried out with the approval of the Ethics Committee of the Affiliated Hospital of Weifang Medical University. All scientific experiments were conducted conforming to World Health Organization guidelines (WHO Laboratory Manual for the Examination and Processing of Human Semen, the 5th edition). Prior written consent was obtained from all males who participated in this study.

### Study population and sample statement

Proteomic analyses were performed on semen obtained from two categories of donors: 7 fertile males who had fathered a child within the last 2 years (control), and 10 males whose spouse had suffered at least two miscarriages (RPL). All males recruited to this study were normozoospermic, in accordance with World Health Organization guidelines. Factors used to exclude patients were abnormal chromosomes, endocrine dysfunction, reproductive duct anomaly, antiphospholipid antibodies, immune disorders or other systemic diseases. Semen samples were ejaculated into sterile containers through masturbation. The samples were processed for 30 min to allow them to liquefy before being centrifuged in a 50% percoll gradient at 1000 g to remove seminal plasma, immature germ cells and nonsperm cells. Afterwards, the samples were frozen at − 80 °C until used.

### Protein preparation and iTRAQ labeling

Proteins were extracted in a 50 mM ammonium hydrogen bicarbonate buffer containing 0.5% sodium deoxycholate, 50 mM dithiothreitol (DTT), and a protease inhibitor. We then sonicated the samples for 1 s (20 times), and centrifuged the samples at 1000 g for 15 min. The supernatant was precipitated overnight with 5vol of acetone and re-suspended in 0.5 M triethylammonium bicarbonate containing 0.5% sodium deoxycholate. Protein concentrations were tested with using the Bradford method [12, 13].

One hundred microgram of protein from each sample was reduced and alkylated, digested with trypsin, dried and reconstituted in 50 μL of 0.5 M triethyl ammonium bicarbonate. In order to avoid inter-individual variations, proteins from all males in each group were pooled in equal quantities. The iTRAQ results were compared between two pool samples. Next, we labeled the dried peptides using an iTRAQ 4-plex kit, in accordance with the AB SCIEX protocol. The protein samples obtained from the control group were labeled with iTRAQ 116, while samples from males of RPL were labeled with 121 iTRAQ reagents. Finally, the labeled samples were mixed in a single vial and dried using a rotary vacuum concentrator [14].

### Liquid chromatography mass spectrometry (LC-MS) analysis

LC-MS analysis was performed on a Triple-TOF 5600 system (AB SCIEX). Mass spectra were collected (400–1250 m/z) at high resolution (> 30,000) for 250 ms per spectrum. A maximum of 50 precursors in a cycle were selected for fragmentation from each mass spectrum. Tandem mass spectra were harvested in high sensitivity mode (resolution> 15,000). 2-plex iTRAQ Labeling, strong cation exchange (SCX) and RP HPLC-MS/MS were performed by Fitgene Biological Technology Co. Ltd. (Guangzhou, China).

### Protein identification and quantitation

Protein identification and quantification were conducted using Protein Pilot Software (AB SCIEX), using the algorithm of Paragon to identify the peptides, which were further analyzed using the Pro GroupTM algorithm in which isoform-specific quantification was employed to track the differences between expressions of different isoforms.

False discovery rate (FDR) was set to less than 0.01 for the identification of both peptides and proteins, and only proteins identified through peptides were used for quantitation. The cutoffod qualification was peptides with an unused confidence score larger than 1.3 and confidence level of 95%. In order to identify differentially expressed proteins, t test was employed to calculate the significant differences in protein expression differences between the RPL and the control group. Proteins with the fold changes of > 1.5 and < 0.67, as well as a *p* value of < 0.05 were identified as the differentially expressed proteins.

### Protein-protein interaction analysis conducted on GeneMANIA

GeneMANIA [15], a user-friendly and flexible web interface for analyzing gene or protein lists, generating hypotheses about gene function, and prioritizing genes for functional assays, was utilized for protein-protein interaction analysis.

Data sets in GeneMANIA were gathered from available and public databases, which include predicted protein interaction data based on orthology from I2D [16]; genetic and physical interaction data from BioGRID [17]; co-expression data from Gene Expression Omnibus (GEO) [18]; and pathway and molecular interaction data from Pathway Commons, which contains data from MINT [19], BioGRID, Reactome [20], HumanCyc [21], IntAct [22], Memorial Sloan-Kettering Cancer Center, Systems Biology Center New York, Human Protein Reference Database [21], and NCI-Nature Pathway Interaction Database [23].

Given a query list, GeneMANIA provides a list of genes or proteins that are functionally similar, or have shared properties with the initial query genes, and displays a functional association network, illustrating the relationships among the list and the curated genomics and proteomics data. The previously identified differentially expressed proteins were added to the search bar, after selecting *Homo sapiens* as the optional organism.

### Protein function and pathway enrichment analysis

In order to better study the biological functions and pathways of differentially expressed proteins, Web-based Gene set analysis toolkit (WebGestalt) was utilized to fully and deeply understand the functional and pathway enrichment information of the interesting proteins [24]. Differentially expressed proteins were uploaded to the WebGestalt server using the overrepresentation enrichment analysis (ORA) method with the Gene Oncology and KEGG databases.

Gene ontology (GO) analysis is a widely used method for analyzing genes and gene products relating to functions including biological processes, molecular functions, and cellular components [25]. Kyoto Encyclopedia of Genes and Genomes pathway (KEGG) is a useful resource for the systematic annotation of gene functions and related high-level genomic functional information [26, 27].

### Protein selection and validation using western blot

Sperm proteins involved in fat and glucose metabolic pathways were selected for validation using western blot. Three key proteins hexokinase-1 (HK1), ATP citrate lyase (ACLY) and fatty acid synthase (FASN) were applied on individual samples obtained from RPL patients (*n* = 10) and the control group (*n* = 7). Proteins from each individual sample of the control and RPL sperm were used for western blot analysis. Primary antibodies: anti-β-actin mouse antibody (Sigma, A5441), anti-HK1 rabbit polyclonal antibody (Abcam, ab150423), anti-ACLY rabbit polyclonal antibody (Abcam, ab40793) and anti-FASN rabbit monoclonal antibody (Abcam, ab128856). Bands were revealed using a chemiluminescence reagent (ECL kit, PerkinElmer, Boston, MA, USA).

## Results

### Protein expression profiling and identification of differentially expressed proteins

**Fig. 1:**
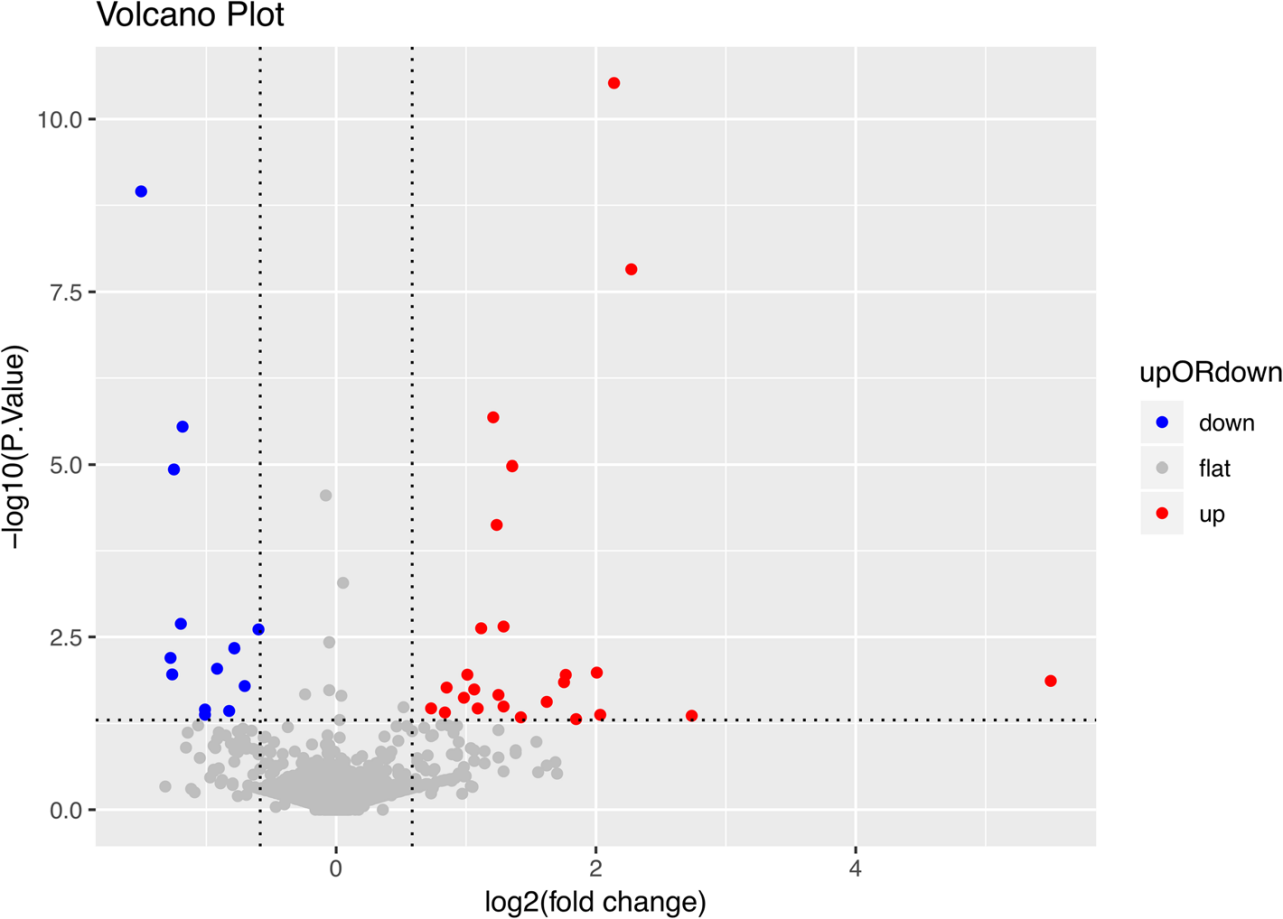
Protein expression profiling and identification of differentially expressed proteins. Volcano plot of differentially expressed proteins. The x axis is the log2 level of protein expression, while the y axis is the log10 level of the *p* value between the RPL and the control group. Each point represents a protein and the different colors denote the differential expression pattern: red for up regulation, grey for flat regulation, and blue for down regulation

Sperm concentration and motility were assessed in the RPL group and the healthy group (Table 1). Statistical analysis showed no difference in sperm concentration and motility between the two groups. Using an iTRAQ based quantitative proteomic method, we identified 2350 proteins in total (Additional file 1: Table S1), and each had peptides with 95% confidence of a minimum unused score of 1.3. The basic statistics of these proteins, such as the isoelectric point, protein mass and peptide number were shown in Additional files 2, 3, 4, 5, 6, 7: Figure S1-S6. Among the 2350 identified proteins, 38 proteins were differentially expressed between the RPL group and the healthy group, with the cutoff fold change of > 1.5 and < 0.67, and a *p* value of < 0.05. To be specific, 25 of these proteins were upregulated, while the other 13 proteins were downregulated in the RPL patients (Fig. 1 and Table 1).

**Table 1:** iTRAQ analysis of differentially expressed proteins between the RPL and the control group

Uniprot ID	Protein Name	Gene Name	Log_2_FC	P value
Up-regulated proteins				
P04279	Semenogelin-1	SEMG1	2.27	1.51E-08
P02751	Fibronectin	FN1	1.24	7.50268E-05
A0A0U1RQF0	Fatty acid synthase	FASN	1.21	2.09185E-06
E7EQB2	Lactotransferrin	LTF	2.14	3.04345E-11
P35579	Myosin-9	MYH9	1.36	1.05659E-05
Q02383	Semenogelin-2	SEMG2	1.62	0.027249441
O75369	Filamin-B	FLNB	0.73	0.033877425
P50991	T-complex protein 1	CCT4	1.06	0.017997051
P53396	ATP-citrate synthase	ACLY	0.84	0.038844213
P35580	Myosin-10	MYH10	1.12	0.002368773
P12277	Creatine kinase B	CKB	1.29	0.002233567
P62258	14–3-3 protein epsilon	YWHAE	1.09	0.033955991
P27487	Dipeptidyl peptidase 4	DPP4	0.85	0.016909158
Q00796	Sorbitol dehydrogenase	SORD	1.77	0.01105386
P12273	Prolactin-inducible protein	PIP	1.75	0.014142492
Q6UX06	Olfactomedin-4	OLFM4	2.03	0.041974943
P49221	Glutamyltransferase 4	TGM4	1.85	0.048483204
P08670	Vimentin	VIM	0.98	0.023722751
O43175	Phosphoglycerate dehydrogenase	PHGDH	2.01	0.01025431
P31947	14–3-3 protein sigma	SFN	1.01	0.011000973
P09525	Annexin A4	ANXA4	1.25	0.021621821
O43776	Asparagine--tRNA ligase	NARS	1.29	0.031847354
P47895	Aldehyde dehydrogenase	ALDH1A3	1.42	0.045620359
X6RBG4	Uromodulin	UMOD	2.74	0.043337822
C9JC07	Myeloid-associated differentiation marker	MYADM	5.50	0.013526945
Down-regulated proteins				
Q5JQC9	A-kinase anchor protein 4	AKAP4	−1.50	1.12E-09
O75969	A-kinase anchor protein 3	AKAP3	−1.25	1.17548E-05
P19367	Hexokinase-1	HK1	−0.60	0.002458935
Q8NEB7	Acrosin-binding protein	ACRBP	−0.82	0.03690489
J3QTJ6	Fibrous sheath-interacting protein 2	FSIP2	−1.18	2.85668E-06
O75976	Carboxypeptidase D	CPD	−0.78	0.004574668
Q8TEX9	Importin-4	IPO4	−1.20	0.002037515
Q8TC56	Protein FAM71B	FAM71B	−0.92	0.00900713
Q9BXF9	Tektin-3	TEKT3	−0.70	0.016060278
A0A0C4DGB6	Serum albumin	ALB	−1.28	0.006274223
A0A0G2JML4	C6orf10	C6orf10	−1.01	0.041954588
Q6ZU69	Protein FAM205A	FAM205A	−1.26	0.010902458
Q9UII2	ATPase inhibitor, mitochondrial	ATPIF1	−1.01	0.035165988

### Protein-protein interaction (PPI) of differentially expressed proteins

**Fig. 2:**
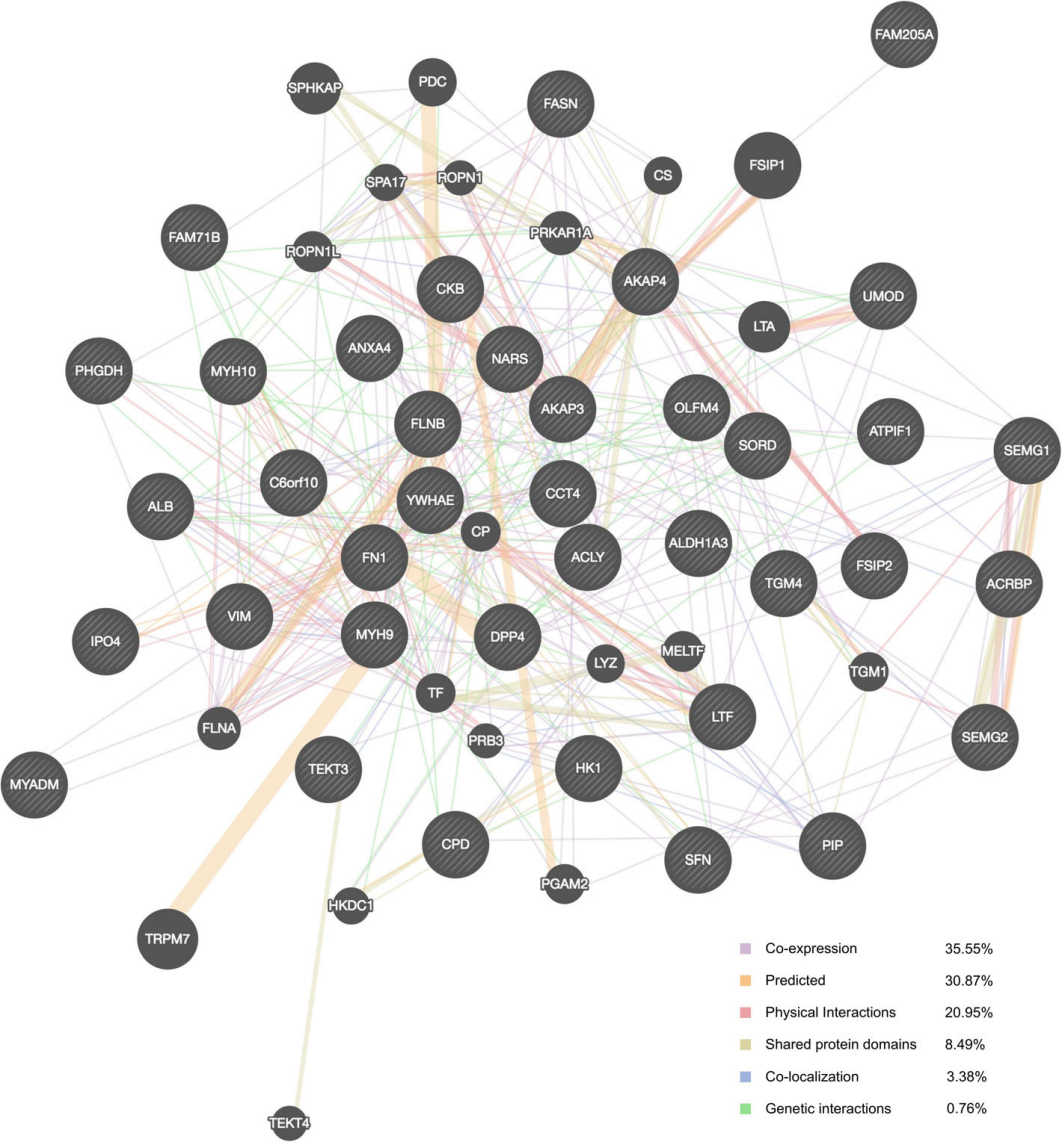
Protein-protein interaction (PPI) network of the differentially expressed proteins. PPI Network of the differentially expressed proteins. Black protein nodes represent target proteins, and the different connecting colors indicate different correlations. Functional association between differentially expressed proteins was investigated using GeneMANIA. Proteins in black circles were the query terms while those in grey circle were indicated proteins

To gain a comprehensive view thorough, we performed a PPI analysis using GeneMANIA. Among the 38 differentially expressed proteins and their interacting proteins, we found that 35.55% displayed similar co-expression, 30.87% were predicted, and 20.95% had physical interaction characteristics. Other results including shared protein domains, co-localization and genetic interaction are shown in Fig. 2.

### Gene ontology enrichment analysis of differentially expressed proteins

**Fig. 3:**
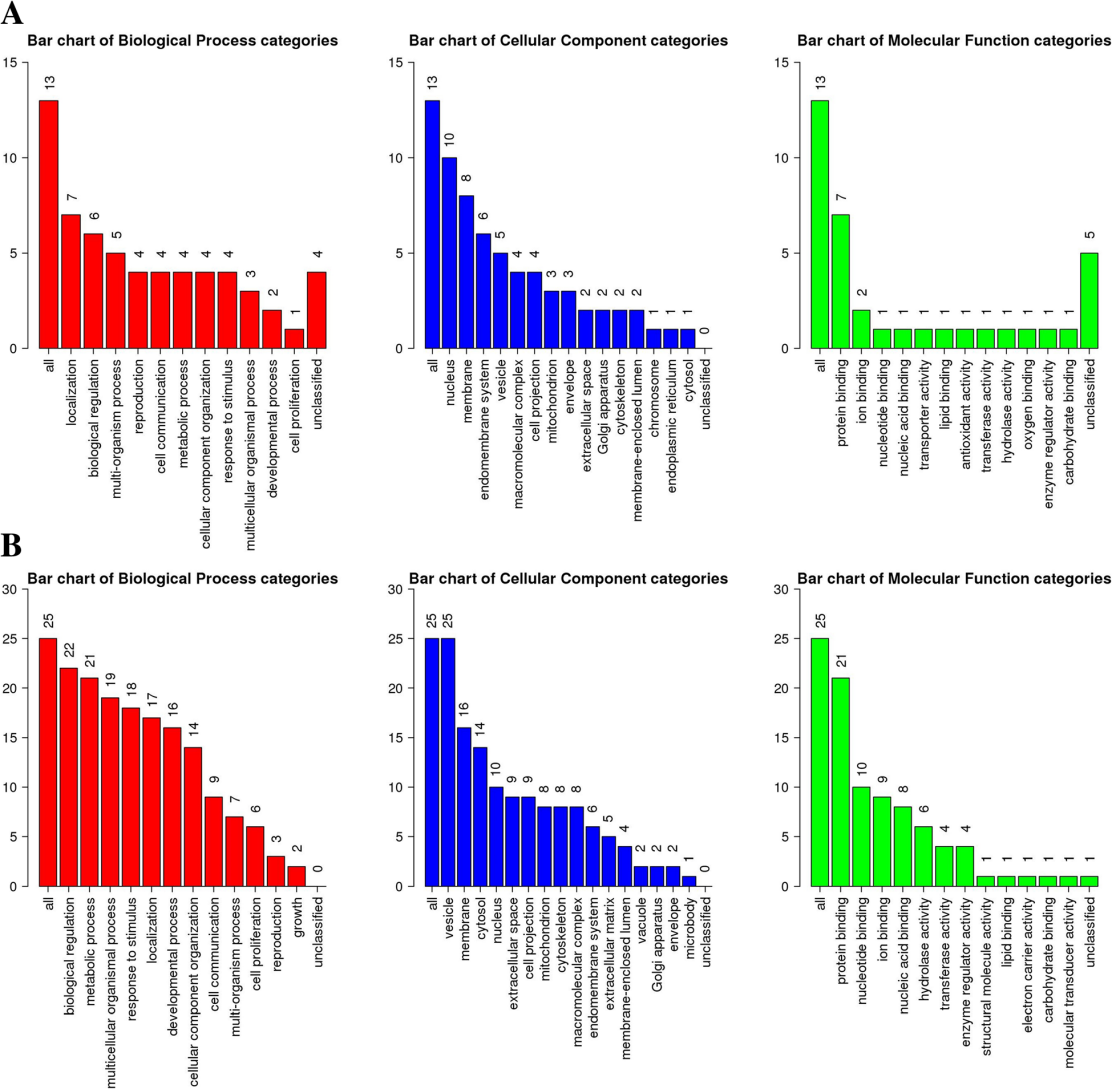
Gene ontology enrichment analysis of differentially expressed proteins. GO enrichment of differentially expressed proteins. **a**: upregulated proteins. **b**: downregulated proteins

In order to understand the functional basis of the 38 differentially expressed proteins, GO enrichment analyses were performed using WebGestalt. The proteins identified were sorted into categories based on the ontology as determined from their GO annotation terms. Interestingly, the differentially expressed proteins were primarily involved in biological regulation, metabolic and cellular processes, protein binding and different enzymes activities, reproduction, and development processes (Fig. 3). Furthermore, most of these proteins were located in vesicle, the nucleuses, membranes and cytosol.

### KEGG pathway enrichment analysis of differentially expressed proteins

**Fig. 4:**
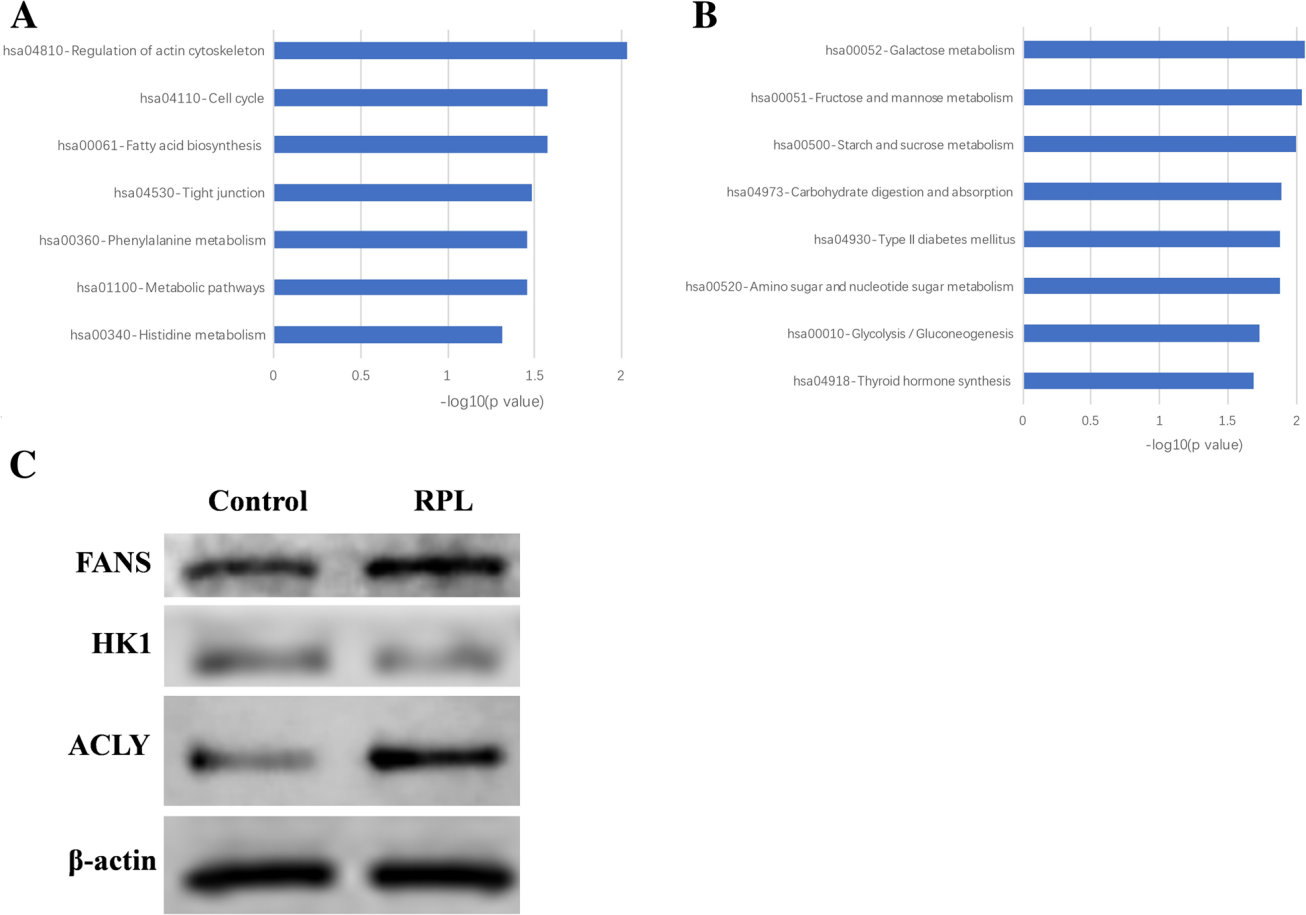
KEGG pathway enrichment analysis of differentially expressed proteins. KEGG enrichment of differentially expressed proteins. **a**: upregulated proteins and **b**: downregulated proteins

To gain insights into the biological pathways of the differentially expressed proteins which were identified through iTRAQ technology, we performed KEGG pathway analyses. Interestingly, it was demonstrated that the upregulated proteins were primarily enriched in fatty acid biosynthesis, while the downregulated proteins were significantly enriched in glucose metabolism (Fig. 4a and b). HK1 was involved in glucose metabolic pathway, while FASN and ACLY were enriched in fat metabolic pathway. So we tested the expressions of the three proteins in RPL samples by western blot (Fig. 4c).

## Discussion

The leading causes for RPL in males are still unknown and the use of the novel proteomic technology may hold the key to more accurately diagnosing and treating male induced RPL [28]. One method of revealing involvement of sperm proteins in the development of RPL is to compare sperm samples from RPL males with samples from healthy individuals.

In this study, we first carried out an iTRAQ proteomic analysis to identify proteins that were differentially expressed between the RPL and the control group. A total of 38 proteins were found to have different expressions between the two groups. It was of great importance to understand why these proteins exerted different expression pattern and how the abnormal expression of the identified proteins could result in male induced RPL. Gene ontology analysis showed that the functions of these proteins could be classified into several important categories, such as biological regulation, metabolic and cellular processes, protein binding and different enzymes activities. More interestingly, we found that differentially expressed proteins were significantly enriched in fatty acid biosynthesis and glucose metabolism pathways. These results demonstrate that disorders of fat and glucose metabolism may contribute to male induced RPL.

Cellular energy metabolism is correlated with cell fate and a few studies report the association between glycogen and the nuclear envelope, the endoplasmic reticulum, as well as the annulate lamellae of embryonic and transformed cells [29, 30]. HK1 protein localizes on the outer membrane of mitochondria and phosphorylates glucose to produce glucose-6-phosphate, which is the first step of glucose metabolism pathways [31]. Spermatogenic cell-specific type 1 hexokinase (HK1S), an isoform of HK1, is found abundantly in sperm, which is mainly expressed in area of the sperm flagellum [32]. HK1 is found to be associated with active spermatogenesis in mice, and might abrogate the process of spermatogenesis leading to infertility [33]. Since glucose metabolism is very important in embryonic development, the downregulation expression of the HK1 might induce miscarriage. Therefore, HK1 functions in RPL need to be further investigated. Furthermore, de novo fatty acid synthesis is activated during embryogenesis, which plays a critical role in embryonic development [34]. FASN is a limited enzyme in fatty acid synthesis, which could catalyze the synthesis of palmitate from acetyl-CoA and malonyl-CoA, in the presence of NADPH [35]. The FASN−/− mutant embryos are died in generated FASN knockout mice [34]. So FASN might play an important role in male induced abortion. Moreover, ACLY is responsible for the synthesis of cytosolic acetyl-CoA in many tissues, and is identified as a potential heat sensitive target in germ cells [36]. The relationship between ACLY and abortion has not been reported, so further research need to be studied.

## Conclusions

We used iTRAQ technology to identify 38 differentially expressed proteins that may be utilized as candidate biomarkers for RPL. The identified proteins and related signaling pathways might play crucial roles in male induced RPL.

## Additional files

Additional file 1: **Table S1.** The total proteins were identified using an iTRAQ based quantitative proteomic method. (XLSX 454 kb)

Additional file 2: **Figure S1.** Isoelectic point distribution. (JPG 783 kb)

Additional file 3: **Figure S2.** Protein mass distribution. (JPG 965 kb)

Additional file 4: **Figure S3.** Peptide number distribution. (JPG 1077 kb)

Additional file 5: **Figure S4.** Distribution of spectral quality matching error. (JPG 2643 kb)

Additional file 6: **Figure S5.** Distribution of proteins’ sequences coverage. (JPG 1699 kb)

Additional file 7: **Figure S6.** Peptide length distribution. (JPG 1118 kb)

## Data Availability

The dataset supporting the conclusions of this article is included within the article.
