# Supplementary figures and images for: Quantitative proteomic analysis of sperm in unexplained recurrent pregnancy loss
Source: Reprod Biol Endocrinol. 2019 Jul 9;17:52. doi: 10.1186/s12958-019-0496-5 (PMC6617596; doi:10.1186/s12958-019-0496-5)

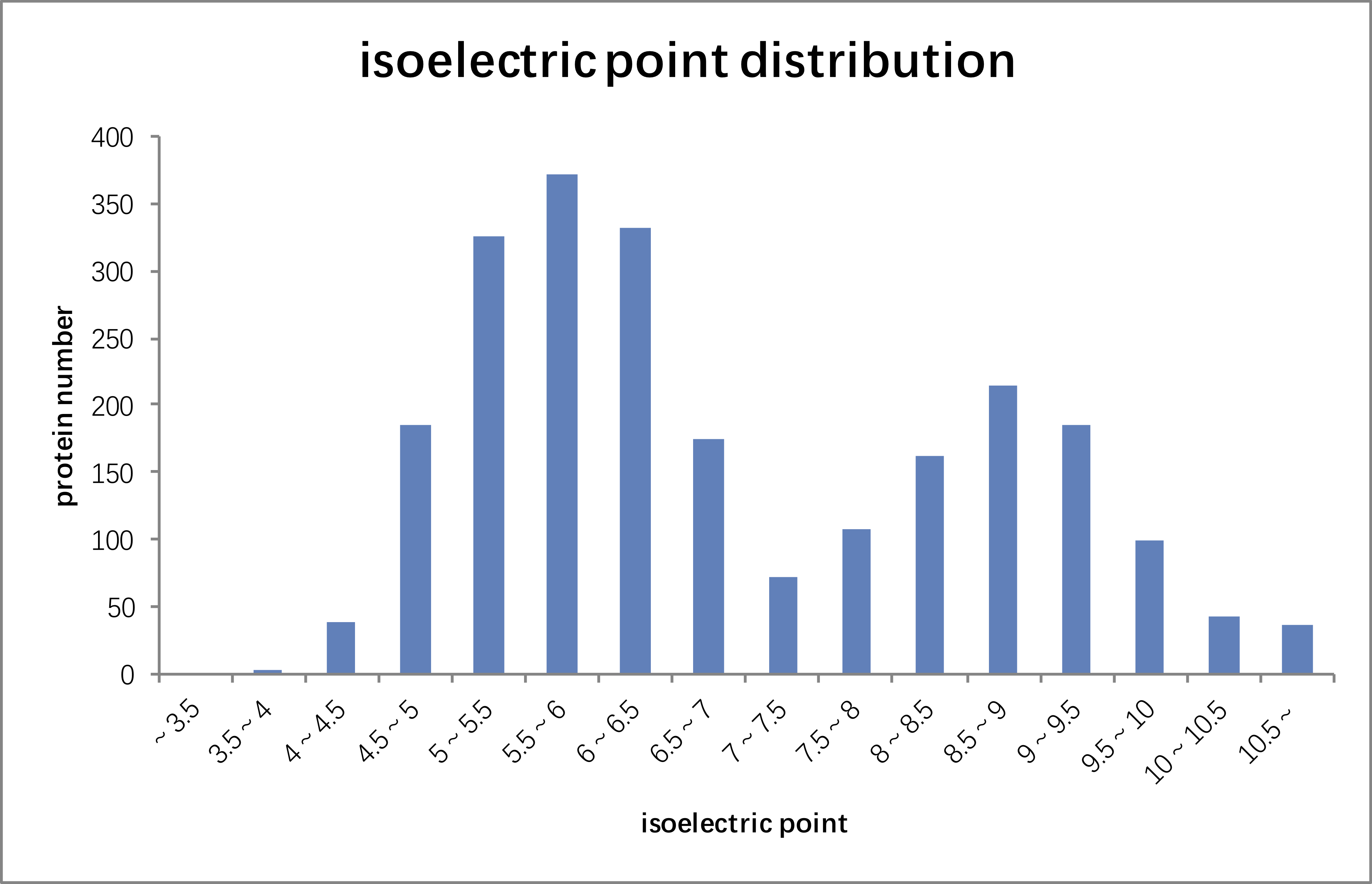

Supplement: Supplementary file 2 — Figure S1. Isoelectic point distribution. (JPG 783 kb) [file 12958_2019_496_MOESM2_ESM.jpg]

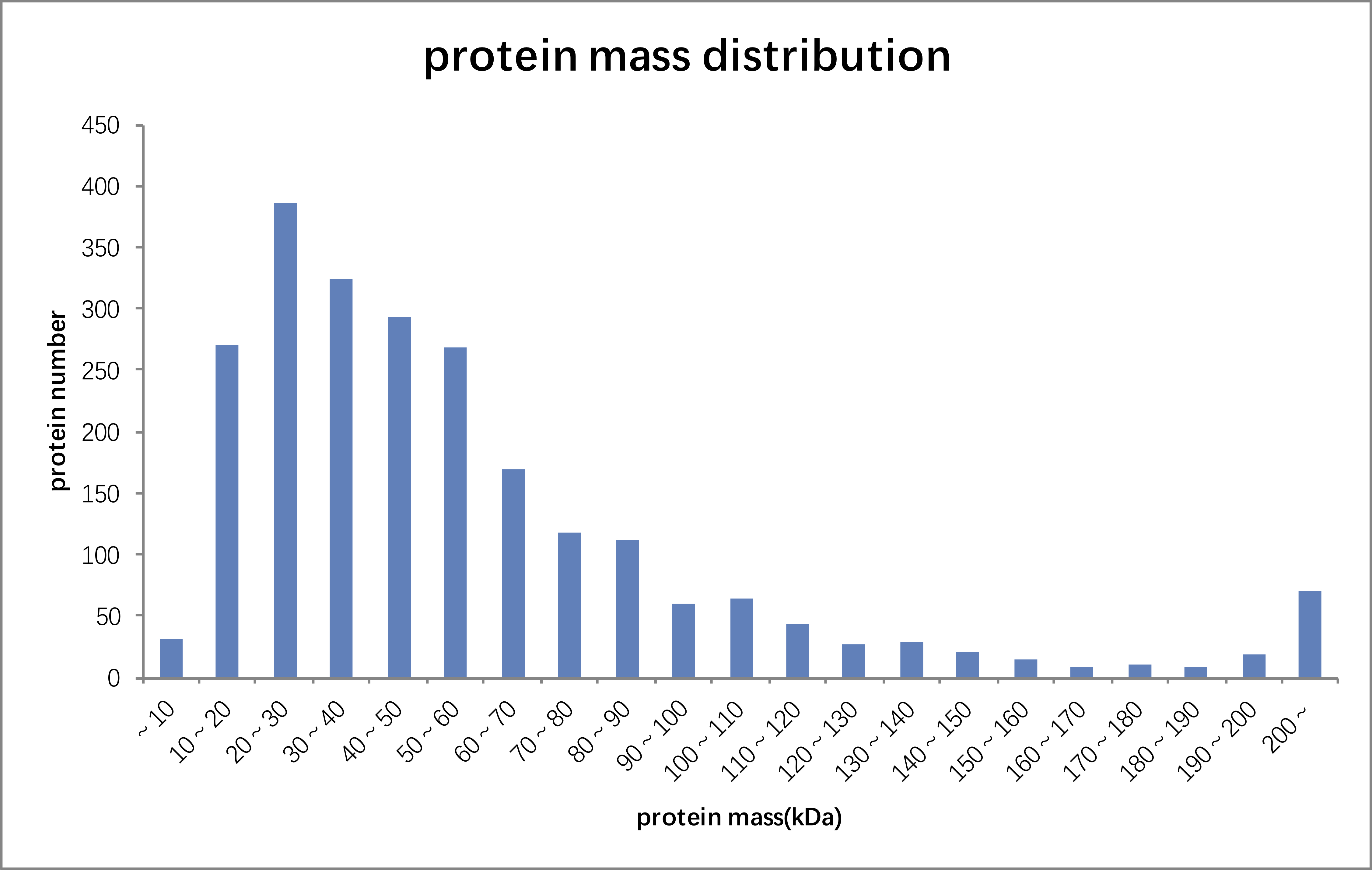

Supplement: Supplementary file 3 — Figure S2. Protein mass distribution. (JPG 965 kb) [file 12958_2019_496_MOESM3_ESM.jpg]

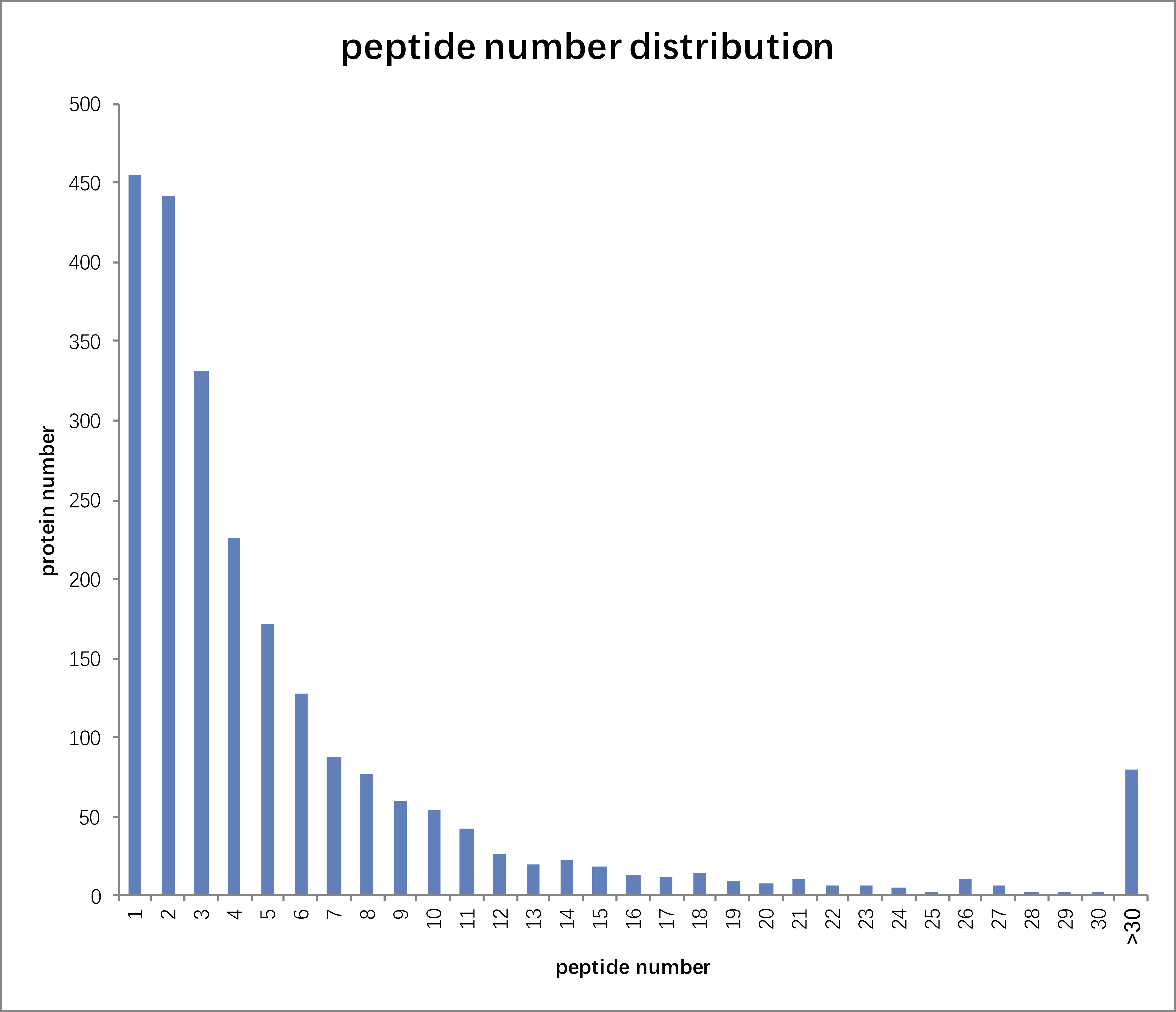

Supplement: Supplementary file 4 — Figure S3. Peptide number distribution. (JPG 1077 kb) [file 12958_2019_496_MOESM4_ESM.jpg]

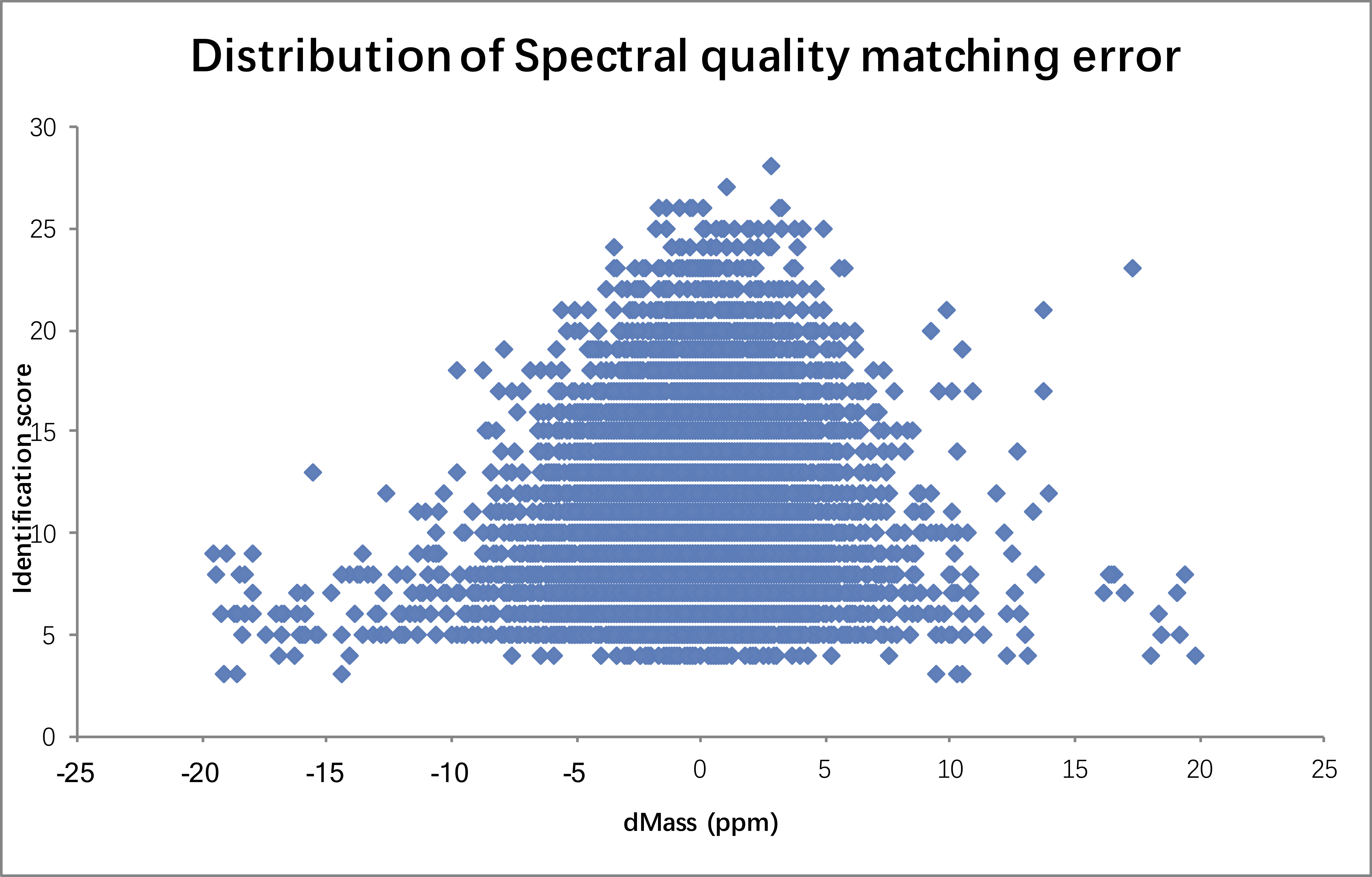

Supplement: Supplementary file 5 — Figure S4. Distribution of spectral quality matching error. (JPG 2643 kb) [file 12958_2019_496_MOESM5_ESM.jpg]

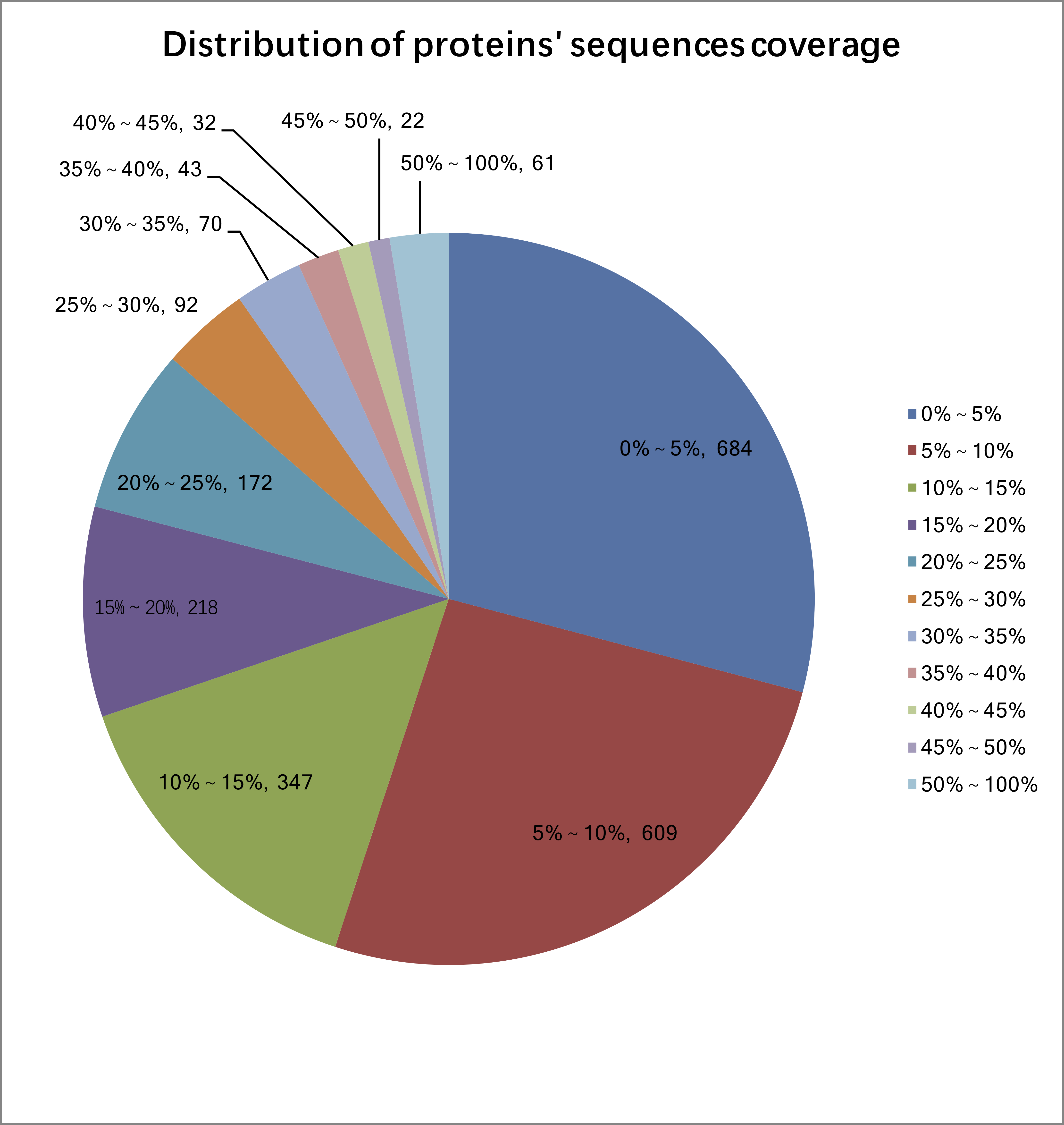

Supplement: Supplementary file 6 — Figure S5. Distribution of proteins’ sequences coverage. (JPG 1699 kb) [file 12958_2019_496_MOESM6_ESM.jpg]

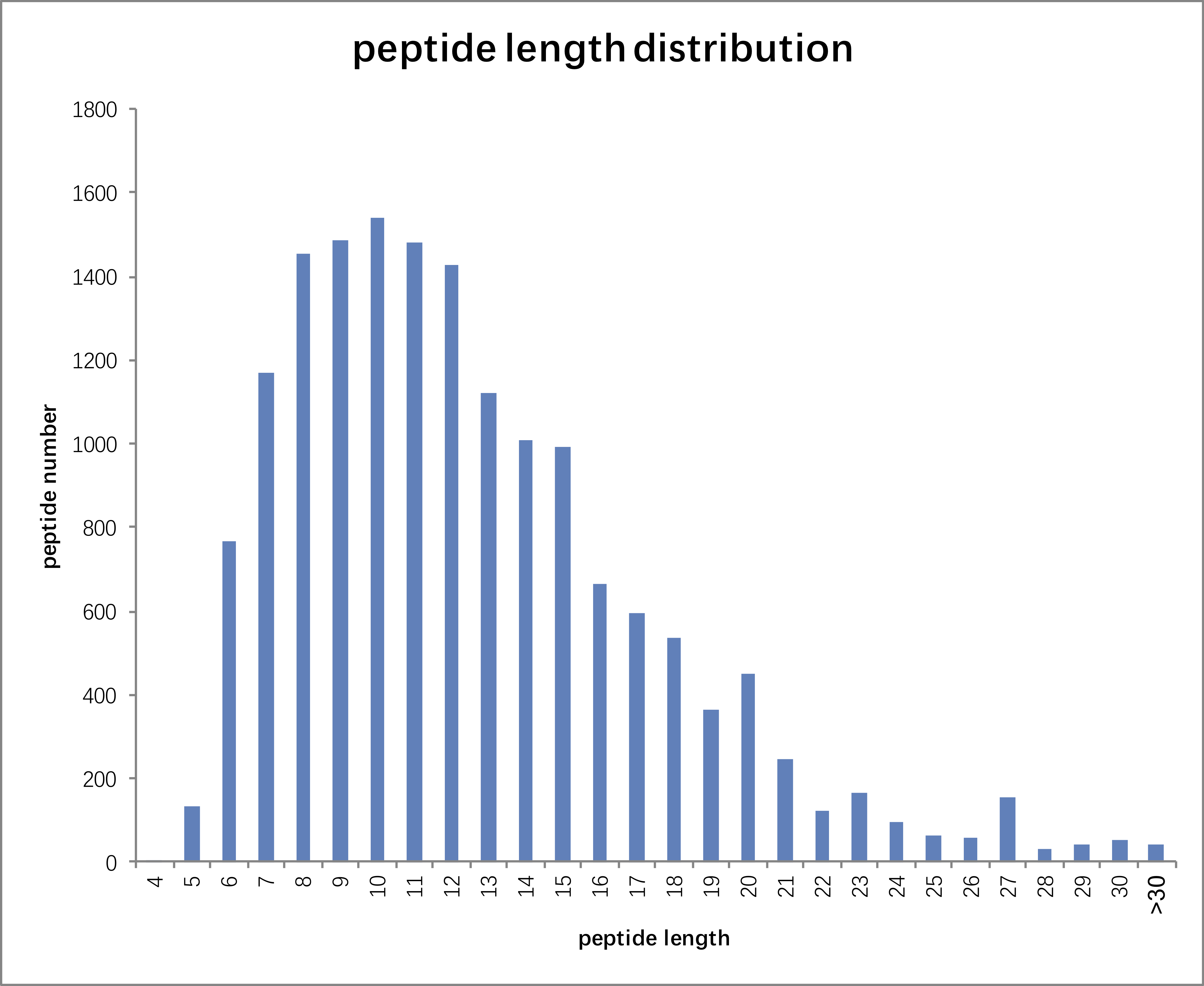

Supplement: Supplementary file 7 — Figure S6. Peptide length distribution. (JPG 1118 kb) [file 12958_2019_496_MOESM7_ESM.jpg]
